# Inclusive algorithmic work design: the moderating role of DEI interventions and diversity-supportive culture in AI-driven workplace transformation

**DOI:** 10.3389/fpsyg.2026.1833850

**Published:** 2026-05-20

**Authors:** Ongeziwe Gift Nodipha, Betty Portia Maphala

**Affiliations:** Department of Industrial and Organizational Psychology, College of Economics and Management Sciences, University of South Africa, Gauteng, Pretoria, South Africa

**Keywords:** artificial intelligence, change readiness, diversity, equity, and inclusion, employee engagement, organisational culture, sociotechnical systems, workplace transformation

## Abstract

Digital transformation has changed how work is designed and governed. Algorithmic management systems and AI tools automate task allocation, performance evaluation, and decision support. While these systems offer efficiency gains, they also risk deepening structural inequalities unless they are embedded in inclusive organisational arrangements. This conceptual paper examines algorithmic work design, organisational culture, and diversity, equity, and inclusion (DEI) interventions. It proposes that DEI-aligned change strategies moderate the link between technology implementation and employee adaptive outcomes. Drawing on sociotechnical systems theory, organisational culture theory, and change readiness frameworks, the paper builds an integrative model that explains how inclusive work design and DEI strategies foster employee trust, psychological safety, and adaptive behaviour during AI-driven transformation. The argument is that DEI interventions moderate the relationship between algorithmic work design and employee outcomes by embedding fairness, transparency, and participation into digital systems. Moreover, a diversity-supportive organisational culture strengthens employees’ readiness for change and their engagement with AI-enabled performance management. By synthesising insights from organisational behaviour, diversity management, and digital work research, the paper provides a novel framework for inclusive digital transformation and offers theoretical propositions and empirical directions for studying equitable AI-mediated workplaces.

## 1 Introduction

The spread of artificial intelligence in workplaces is one of the largest organisational changes since the industrial revolution. Organisations in many sectors now use AI systems to automate decisions, assign tasks, evaluate performance, and manage workers (Kellogg et al., 2020; Newell and Marabelli, 2015; Raisch and Krakowski, 2021). These algorithmic management tools promise better efficiency, objective decisions, and data driven insights. Yet they also raise serious questions about fairness, transparency, employee autonomy, and the possible reproduction of existing inequalities (Ajunwa, 2020; Jarrahi and Sutherland, 2019; Rana and Awan, 2024).

The intersection of algorithmic work design and diversity, equity, and inclusion has become a key research area. Studies show that AI can perpetuate and even amplify biases, especially when training data contain historical patterns of discrimination (Benjamin, 2019; Buolamwini and Gebru, 2018; Noble, 2023). Also, how employees react to algorithmic management, whether they accept, adapt, resist, or disengage, depends heavily on how the systems are designed, communicated, and integrated into daily work (Brynjolfsson and McAfee, 2014; Colbert et al., 2016; Bankins and Formosa, 2023). Understanding what shapes these reactions is essential for organisations that want to use AI while keeping workplaces fair and inclusive.

Organisational culture plays a decisive role. Cultures that value diversity, support psychological safety, and encourage employee participation tend to produce positive responses to technological change. In contrast, cultures built on hierarchy, surveillance, and low transparency worsen anxieties about algorithmic control (Cameron and Quinn, 2011; Schein, 2010; Hartnell et al., 2024). At the same time, deliberate DEI interventions can act as moderators: they can buffer negative effects of algorithmic systems and strengthen positive ones.

This conceptual paper develops an integrated framework that brings together algorithmic work design, organisational culture, and DEI interventions. Using sociotechnical systems theory, organisational culture theory, and change readiness frameworks, the authors propose a model that shows how inclusive work design and DEI aligned strategies foster trust, psychological safety, and adaptive behaviour during AI driven change. The paper makes three distinct contributions. First, it is the first framework to position DEI interventions as explicit moderators within a sociotechnical systems analysis of AI implementation, rather than treating DEI as a peripheral or downstream issue. Second, it conceptualises organisational culture not as a static background but as a reciprocal factor: culture shapes AI implementation, and AI implementation reshapes culture (Proposition 5). Third, the paper provides a theoretical stress test and a concrete empirical operationalisation roadmap, moving beyond abstract propositions to testable designs. These contributions distinguish the framework from existing work on algorithmic fairness, inclusive design, and sociotechnical systems.

## 2 Theoretical foundation

### 2.1 Sociotechnical systems theory

AI implementation requires frameworks that capture the mutual shaping of technology and social dynamics. Trist and Bamforth (1951) sociotechnical systems theory, later refined by Pasmore et al. (2018) and Davis et al. (2022), holds that organisational performance emerges from the joint optimisation of technical and social systems. Neither domain can be optimised in isolation. This is especially relevant for algorithmic management, where AI reconfigures tasks and interpersonal relations at the same time.

Three principles guide the present framework. First, joint optimisation means AI systems should be designed not only for technical efficiency but also for compatibility with human needs, capabilities, and social interactions (Cherns, 1976; Clegg, 2000; Hinds and Cramton, 2024). Second, boundary management requires careful attention to interfaces between technical and social subsystems to avoid friction (Rice, 1958). Third, participation matters: employees who are involved in designing and implementing technological systems show higher acceptance and more effective use (Mumford, 2006; Schmiedel et al., 2023).

### 2.2 Organisational culture theory

Schein (2010) layered model and Cameron and Quinn (2011) competing values framework are standard references, but they assume a level of cultural stability that AI may undermine. AI can simultaneously disrupt visible artefacts, espoused values, and deep assumptions about expertise and autonomy (Vial, 2023). Therefore, this paper combines cultural theory with sociotechnical thinking, treating AI implementation as a cultural intervention that renegotiates taken for granted beliefs about human judgement.

Culture operates at multiple levels, from observable behaviours to underlying assumptions about work, relationships, and effectiveness. Research on technology implementation shows that cultures emphasising flexibility, innovation, and development produce better responses to change than those prioritising stability and control (Shin and Lee, 2022). Psychological safety the shared belief that the team is safe for interpersonal risk taking, is especially important. It enables employees to voice concerns, experiment with new technologies, and adapt (Edmondson, 1999; Edmondson and Bransby, 2023). Recent work in Frontiers in Sociology (2024) stresses that AI-driven change does not just happen within an existing culture; it actively reshapes that culture over time, requiring a dynamic view.

### 2.3 Change readiness frameworks

**Figure 1:**
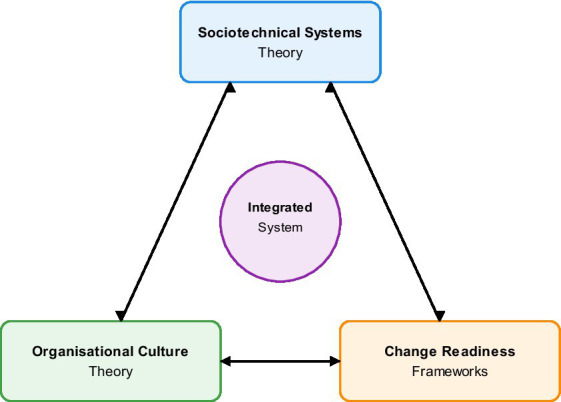
Integration of theoretical foundations.

Change readiness frameworks explain why some employees are prepared and willing to engage with organisational transformations while others are not (Armenakis and Harris, 2009; Weiner, 2009; Vakola, 2021). Readiness includes cognitive appraisals of the change, affective responses, and behavioural intentions. Key antecedents are perceived need for change, appropriateness of the change approach, organisational support, and change efficacy (Rafferty and Simons, 2006; Madsen et al., 2023). In the context of AI, these antecedents are shaped by how algorithmic systems are designed, communicated, and integrated. DEI considerations are integral here because employees from different backgrounds may have different perceptions of algorithmic management based on prior experiences with organisational systems (Trittin-Ulbrich and Scherer, 2024; Figure 1).

## 3 Conceptual model and propositions

### 3.1 A typology of algorithmic work design

Algorithmic work design refers to how tasks, roles, and decision processes are configured by or mediated by AI systems (Kellogg et al., 2020; Meijerink and Bondarouk, 2023). Because different systems have different effects, the authors propose a typology along three axes:

- Decision autonomy—advisory (AI suggests, human decides), prescriptive (AI recommends and human must justify deviation), fully automated (AI decides without human intervention).
- Functional domain—task allocation, performance evaluation, scheduling, monitoring, or decision support.
- Transparency potential—inherently interpretable (e.g., decision trees) versus black box (e.g., deep neural networks).

The propositions below are qualified by this typology. For example, the positive effects of transparency are strongest for advisory systems and for high stakes domains like performance evaluation; for fully automated black box systems, other governance mechanisms become critical.

### 3.2 Algorithmic work design and employee outcomes

Characteristics of algorithmic work design directly influence employee trust, job satisfaction, engagement, and adaptive behaviour. Transparent and explainable systems build trust because workers can understand decisions and perceive the system as fair (Dietvorst et al., 2015; Lee and See, 2004; Langer and Landers, 2024). Opaque “black box” systems breed suspicion and resistance (Glikson and Woolley, 2020; Bankins et al., 2024).

However, transparency is not a free good. There is a well-known trade-off between predictive accuracy and explainability (Rudin, 2019; Arrieta et al., 2020; Vilone and Longo, 2021). Deep learning models may be very accurate but hard to explain, while simple models are easier to interpret but may be less accurate. Organisations must navigate this trade off contextually, prioritising transparency in high risk decisions (e.g., hiring, promotion) while accepting lower transparency in low risk optimisation tasks.

Proposition 1. Algorithmic work configurations that maintain procedural transparency, meaningful explainability, and employee autonomy are positively associated with employee trust, job satisfaction, and adaptive behaviour during technological transition, provided that the transparency does not overwhelm employees with irrelevant information (i.e., it must be actionable and user friendly). This effect is strongest for advisory and prescriptive systems in high stakes domains.

### 3.3 The moderating role of DEI interventions

DEI interventions are deliberate, time bound organisational actions: bias audits, inclusive design processes, algorithmic literacy training, grievance mechanisms for unfair treatment, and accountability structures (Kalev et al., 2006; Dobbin and Kalev, 2016; Frontiers in Psychology, 2024). They moderate the relationship between algorithmic work design and employee outcomes. When robust DEI interventions are in place, the negative effects of algorithmic management are reduced and the positive effects amplified. Mechanisms include bias auditing that improves fairness perceptions (Raji et al., 2020; Dokuka et al., 2023); training that reduces anxiety and builds confidence (Tambe et al., 2019; Voronov and Urick, 2024); and participatory design that gives employees voice and ownership (Fehrer et al., 2024).

Yet DEI interventions can fail or backfire. Mandatory diversity training may provoke reactance; bias audits without enforcement become performative; grievance channels that are not trusted may deter reporting (Kalev et al., 2006; Dobbin and Kalev, 2016). The following propositions therefore include boundary conditions.

Proposition 2a. DEI interventions moderate the relationship between algorithmic work design and employee outcomes: the positive effects of well-designed systems are strengthened, and the negative effects of poorly designed systems are weakened, when DEI interventions are implemented with fidelity, accompanied by accountability mechanisms, and perceived by employees as genuine rather than tokenistic.

Proposition 2b. The effectiveness of DEI interventions is itself moderated by the existing diversity supportive culture: in cultures that are already inclusive, DEI interventions have stronger positive effects; in hostile cultures, they may produce backlash unless combined with leadership commitment and structural changes.

Proposition 2c. Under conditions of “DEI fatigue” or when interventions are perceived as compliance driven rather than justice oriented, the moderating effect may become null or even negative.

### 3.4 Organisational culture: distinguishing DEI interventions from diversity-supportive culture

**Proposition 3.** Diversity-supportive organisational culture is positively associated with employee change readiness and engagement with AI-enabled systems. This effect operates partly by enhancing the perceived legitimacy of DEI interventions and partly by creating a psychological climate in which algorithmic transparency is expected and valued.**Proposition 5 (reciprocal influence).** The implementation of AI systems reciprocally influences organisational culture. Transparent, fair, and participatively designed AI can over time strengthen diversity-supportive cultural attributes. Conversely, opaque, biased, or imposed AI can erode trust and inclusiveness, even in previously supportive cultures.

### 3.5 Psychological safety as a mediator

Psychological safety, the belief that the workplace is safe for interpersonal risk-taking, mediates the relationships between the antecedents and employee outcomes. The mechanisms are threefold:

- *Cognitive pathway:* Transparency and explainability reduce uncertainty, helping employees build accurate mental models of how AI works.
- *Affective pathway*: Perceived fairness and inclusive processes lower anxiety, freeing cognitive resources for learning and adaptation.
- *Behavioural pathway*: Psychological safety encourages voice, experimentation, and feedback-seeking, all of which are necessary for effective use of new AI-mediated routines (Edmondson and Bransby, 2023; Kessel et al., 2022).

**Proposition 4.** Psychological safety mediates the relationships between (a) algorithmic work design, (b) DEI interventions, (c) diversity-supportive culture, and employee adaptive behaviour. This mediation is stronger when employees have adequate algorithmic literacy and when they perceive that speaking up will not lead to retribution.

### 3.6 Moderated mediation framework

**Figure 2:**
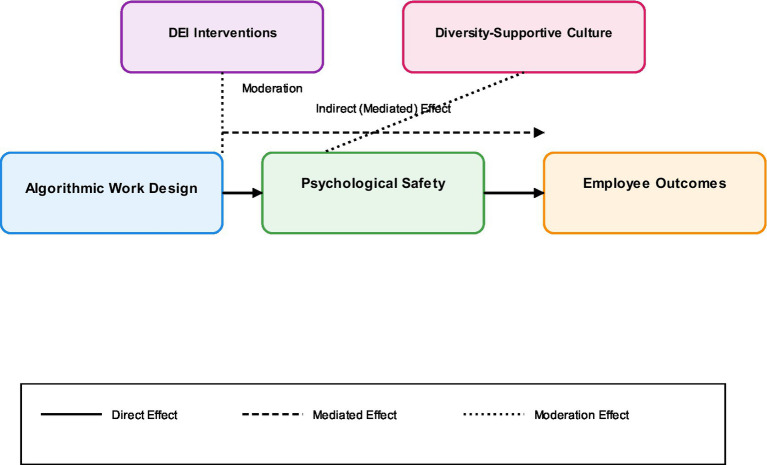
Moderated mediation conceptual model.

The model is explicitly a moderated mediation model. Algorithmic work design affects employee outcomes partly *through* psychological safety (mediation), and the strength of that mediated path is moderated by DEI interventions and by diversity-supportive culture (moderated mediation). Figure 2 represents this.

## 4 Discussion

### 4.1 Theoretical contributions

The paper makes four contributions. First, it offers the first explicit integration of DEI interventions as *moderators* in a sociotechnical framework for AI implementation. Prior work has discussed DEI as an outcome or a parallel concern, not as a lever that changes how algorithmic design affects employees. Second, it introduces a dynamic, reciprocal view of organisational culture (Proposition 5), responding to critiques that traditional models assume too much stability. Third, it provides a typology of algorithmic work design and a moderated mediation specification, increasing analytical precision. Fourth, it includes a theoretical stress test and an empirical operationalisation roadmap, moving beyond abstract propositions.

### 4.2 Human-AI relational dynamics

AI systems change relational roles. Algorithms may mediate between managers and subordinates, increase relational distance, and introduce new forms of accountability (Jarrahi and Sutherland, 2019; Murray et al., 2024). When AI takes on evaluative functions, relational trust can erode unless the system is designed to augment human judgement rather than replace it (Glikson and Woolley, 2020). DEI interventions that include contestability mechanisms (e.g., appeals processes) can restore relational agency (Bankins and Formosa, 2023). Organisations should design not only for task efficiency but also for relational quality.

### 4.3 Theoretical stress test

Three foreseeable technological developments challenge or extend the model.

- Generative AI and agentic systems—Employees may shift from being *supervised* by AI to being *collaborators* with AI (Raisch and Krakowski, 2021). Transparency becomes harder because generative models are often less interpretable. The model’s propositions about explainability would need to be supplemented with new concepts like “contestability” and “alignment.”
- Predictive and pre-emptive analytics—AI that predicts future performance or attrition can create anticipatory control. Bias in predictive models may disproportionately affect minority groups (Cech and Waidzunas, 2021). The moderating role of DEI interventions becomes even more critical, but the model would need specific propositions about the timing and framing of predictions.
- Biometric and affective computing—Emotional and physiological data raise privacy and consent issues. Psychological safety becomes paramount, as employees may fear surveillance beyond task performance (Rana and Awan, 2024). The model’s reliance on transparency may need to be extended to include explicit consent and data governance.

The core propositions are centred on fairness, participation, transparency, and psychological safety—remain resilient, but their operationalisation must adapt. Future research should test the model across these scenarios.

### 4.4 Towards empirical operationalisation

To make the framework testable, the authors suggest concrete measures.

- Algorithmic transparency: Use the scale from Lee and See (2004) and Dietvorst et al. (2015), supplemented by objective checks (e.g., does the system provide user-understandable explanations?).
- DEI intervention intensity: A composite index counting the presence of bias audits, inclusive design processes, algorithmic literacy training, grievance mechanisms, and accountability structures, weighted by perceived quality (cf. Dobbin and Kalev, 2016; Kordzadeh and Ghasemaghaei, 2022).
- Diversity-supportive culture: The Diversity Climate Scale (Dwertmann et al., 2016) or qualitative coding of policies and interview data (Quigley and Dutton, 2023).
- Psychological safety: Edmondson (1999) scale, updated by Edmondson and Bransby (2023).
- Employee adaptive behaviour: Self-reported change readiness (Rafferty and Simons, 2006; Madsen et al., 2023) combined with objective indicators like system usage or peer ratings.

Longitudinal, mixed-methods designs (surveys before and after AI implementation, plus interviews) can test causality and capture cultural change as proposed in Proposition 5.

### 4.5 Boundary conditions and potential counter-effects

The positive effects proposed are not universal. Four boundary conditions are especially important.

1. Algorithmic literacy: Without basic literacy, even transparent systems may confuse employees, weakening the mediation through psychological safety.
2. Institutional trust: In organisations with a history of broken promises, DEI interventions may be met with cynicism.
3. Job autonomy baseline: In highly routinised jobs, giving autonomy may be less relevant than in knowledge work.
4. Cultural context: In high-power-distance cultures, employees may be less willing to voice concerns even when psychological safety is nominally present (Leidner and Kayworth, 2024).

Also, as noted, DEI interventions can backfire. Mandatory training without follow-up can increase resistance. Bias audits that are not acted upon can deepen distrust. The manuscript now includes a dedicated “Caveats” paragraph in Section 4.6 (Practical Implications).

### 4.6 Practical implications

Organisations should take four actions.

First, design for transparency and explainability, but tailor the level to the domain and user. For high-stakes decisions, use interpretable models or provide clear post-hoc explanations. Train employees to interpret system outputs.

Second, invest in DEI interventions that are substantive, not symbolic. Bias audits must lead to system changes. Training should be voluntary, continuous, and combined with structural accountability (e.g., diversity metrics in managerial incentives). Grievance mechanisms must be confidential, accessible, and trusted.

Caveats: Mandatory diversity training has been shown to sometimes reduce diversity (Dobbin and Kalev, 2016). Bias audits can be performative if no remediation follows. Therefore, the authors recommend that DEI interventions be accompanied by (a) leadership commitment, (b) third-party validation, and (c) iterative feedback loops.

Third, cultivate a diversity-supportive culture as a foundation. This includes inclusive leadership development, equitable policies, and protection for employees who raise concerns about algorithmic fairness.

**Figure 3:**
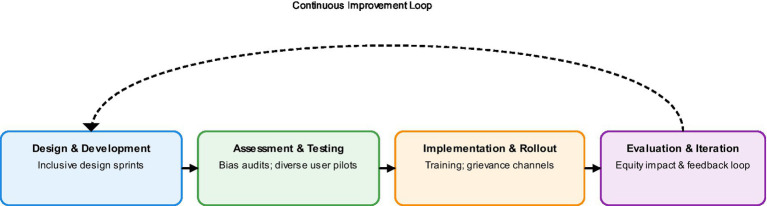
Inclusive Al implementation process.

Fourth, build psychological safety deliberately. Leaders should model openness, create safe forums for discussing AI-related anxieties, and ensure that speaking up leads to constructive action, not punishment (Edmondson and Bransby, 2023; Figure 3).

### 4.7 Implications for global south contexts

The framework is largely universal, but Global South contexts introduce specific challenges and opportunities. In many low- and middle-income countries, digital infrastructure is uneven, regulatory protections for workers are weaker, and historical inequalities intersect with new algorithmic systems (Rana and Awan, 2024). For example, in South Africa, the legacy of apartheid labour practices means that performance algorithms trained on historical data may reproduce racial disparities in promotion. Moreover, data colonialism—where data are extracted from vulnerable populations without fair benefit—is a real risk. Culturally appropriate DEI interventions must be co-designed with local communities, not imported wholesale from Western contexts. The authors, based in South Africa, encourage future research to adapt the model to different institutional and cultural settings.

### 4.8 Future research directions

Quantitative studies should test the moderated mediation model using longitudinal surveys. Qualitative case studies can explore the lived experience of employees, especially in non-Western settings. Cross-sectoral and cross-cultural comparisons (e.g., healthcare vs. manufacturing; collectivist vs. individualist cultures) would clarify boundary conditions. Finally, researchers should study long-term DEI outcomes: does inclusive algorithmic design actually improve representation and pay equity over time?

### 4.9 Limitations

As a conceptual paper, the framework requires empirical validation. It focuses on individual-level outcomes and may miss team or organisational dynamics. The rapid evolution of AI means that some specifics (e.g., types of transparency) may need updating. Also, the framework assumes that DEI interventions can be implemented with reasonable fidelity; conditions of state-captured or highly corrupt organisations may require a separate model. These limitations are acknowledged, and the authors invite empirical testing and refinement.

## 5 Conclusion

The integration of AI into workplace processes is a profound transformation. It forces organisations to balance efficiency with equity, transparency, and employee well-being. This paper has presented an integrative framework showing how algorithmic work design, DEI interventions, and organisational culture interact to shape employee responses to AI-driven change. By embedding DEI considerations into the design and implementation of algorithmic systems, organisations can foster the trust, psychological safety, and adaptive behaviour needed for successful digital transformation.

The author’s position in South Africa shapes this analysis. In contexts where inequality is deeply embedded, the choices about how AI is deployed are not merely technical but moral. The framework is intended to be globally relevant, but its principles of participation, transparency, and accountability are especially urgent where trust in institutions is fragile. As AI continues to evolve, the imperative for inclusive algorithmic work design will only grow. This paper provides a foundation for scholarly inquiry and practical action towards more equitable AI-mediated workplaces.
